# Fabrication of Paper-Based Microfluidics by Spray on Printed Paper

**DOI:** 10.3390/polym14030639

**Published:** 2022-02-08

**Authors:** Yi-Je Juang, Shu-Kai Hsu

**Affiliations:** 1 Department of Chemical Engineering, National Cheng Kung University, No. 1 University Road, Tainan 70101, Taiwan; rfpjhnmf@gmail.com; 2 Center for Micro/Nano Science and Technology, National Cheng Kung University, No. 1 University Road, Tainan 70101, Taiwan; 3 Research Center for Energy Technology and Strategy, National Cheng Kung University, No. 1 University Road, Tainan 70101, Taiwan

**Keywords:** microfluidics, spray, printing paper, polydimethylsiloxane, glucose detection

## Abstract

Since the monumental work conducted by Whitesides et al. in 2007, research and development of paper-based microfluidics has been widely carried out, with its applications ranging from chemical and biological detection and analysis, to environmental monitoring and food-safety inspection. Paper-based microfluidics possesses several competitive advantages over other substrate materials, such as being simple, inexpensive, power-free for fluid transport, lightweight, biodegradable, biocompatible, good for colorimetric tests, flammable for easy disposal of used paper-based diagnostic devices by incineration, and being chemically modifiable. Myriad methods have been demonstrated to fabricate paper-based microfluidics, such as solid wax printing, cutting, photolithography, microembossing, etc. In this study, fabrication of paper-based microfluidics was demonstrated by spray on the printed paper. Different from the normally used filter papers, printing paper, which is much more accessible and cheaper, was utilized as the substrate material. The toner was intended to serve as the mask and the patterned hydrophobic barrier was formed after spray and heating. The processing parameters such as toner coverage on the printing paper, properties of the hydrophobic spray, surface properties of the paper, and curing temperature and time were systematically investigated. It was found that, after repetitive printing four times, the toner was able to prevent the hydrophobic spray (the mixture of PDMS and ethyl acetate) from wicking through the printing paper. The overall processing time for fabrication of paper-based microfluidic chips was less than 10 min and the technique is potentially scalable. Glucose detection was conducted using the microfluidic paper-based analytical devices (µPADs) as fabricated and a linear relationship was obtained between 1 and 10 mM.

## 1. Introduction

As the first microscale gas chromatography system was manufactured in 1979, it heralded the beginning of microfluidics as a field of its own [1]. Since then, utilization of microfluidic platform for numerous applications ranging from point-of-care diagnostics, rapid test, forensics, food-safety inspection, environmental monitoring, and biotechnology has been extensively explored [2,3,4,5,6,7,8]. Compared to the conventional analytical approaches, microfluidics provides several merits such as fast response, high throughput, low reagent consumption, reduced waste product, high sensitivity, and great portability. Although different substrate materials such as silicon, glass, polymer, and elastomer [9,10] have been exploited to construct the microfluidic devices, paper stands out as an attractive alternative because it is low-cost and ubiquitous [11]. In addition, its hydrophilicity and porous nature make reagent storage feasible and enable power-free fluid transport via the capillary [12]. Moreover, paper is biodegradable, biocompatible, and flammable for easy disposal by incineration and good for colorimetric tests [13]. The use of a paper device was first demonstrated by Müller and Clegg dating back to 1949 [14], however, it was not until 2007 that paper-based microfluidics regained attention, thanks to the work conducted by Whitesides’s group.

For paper-based microfluidics, the most common and extensively used paper substrate is cellulose-based paper [15]. Since this type of paper possesses great wicking ability, relatively uniform thickness, and superior adsorption and retention of reagents [16], paper chips made of Whatman #1, #3, and #5 filter paper, tomographic paper, and chromatography paper have been used in health, food, and environmental and biological detection. Different from cellulose-based paper, nitrocellulose membranes have chemical functional groups such that the biomolecules can be covalently immobilized on the paper [16]. This leads to their high protein-binding abilities, which are suitable for enzyme-linked immunosorbent assays (ELISA) and gold nanoparticle-based assays. Paper towel is cheaper than filter paper and highly porous, and attempts have been made to screen-print electrodes on the paper towel [17] or use it as a wicking paper in the paper chip [18]. Owing to its unique properties of being strong, sustainable, waterproof, and tear-resistant, mineral paper has been adopted to construct a paper-based capillary electrophoresis microdevice for pathogen detection [19]. The glass fiber membranes are biochemically/chemically inert and possess excellent mechanical, electrical, and electroosmosis properties. Therefore, attempts have been made to fabricate paper-based microfluidics for urine bioassays [20] and colorimetric analyte detection in whole blood [21]. In order to anneal silver nanoparticle electrode arrays on the paper substrate at high temperature, inkjet photo paper was selected as the substrate because it is glossy and thermally stable up to 200 °C [22]. Task wipers were also used, for their high wicking speed [23].

As for fabrication of paper-based microfluidics, this can be divided into two groups, that is, patterning the hydrophobic barrier and shaping [14]. For the former, it can be further organized into four categories: plotting, printing, masking, and stamping [24]. As to the latter, cutting, embossing, and laser etching are included [14]. The pros and cons for each method are summarized in the literature [14]. For example, the photolithographic technique was first adopted by Whitesides’s group and the channel wall, which is the solid, hydrophobic barrier, is the cured photoresist. The advantages of this approach are high resolution and high throughput; however, expensive equipment and reagents are required and the potential contamination of the channel is concerned. Wax printing is the most popular and widely used technique owing to its high throughput, low cost, and simple fabrication. The barrier is formed through first depositing the wax on the filter paper by a wax printer, followed by melting and solidifying the melted wax. The resolution is influenced by the heating temperature and types of the filter paper [25] and the processing time varies depending on the complexity of the microfluidic design. In most cases, the heating temperature is above 100 °C in order to shorten the heating time to approximately 3–5 min. One of the key ingredients of the wax printing technique is the solid wax printer, which unfortunately is no longer in production after 2017. Stamping is also a simple and low-cost technique which transfers hydrophobic materials (e.g., paraffin, ink, PDMS, etc.) into filter paper by contacting the stamp with the filter paper and subsequently forming the barrier [26,27,28]. The resolution of the device is relatively low and a mask for each pattern is required. Spraying is another simple and low-cost alternative for fabrication of paper-based microfluidics [29,30]. Nurak et al. sandwiched the filter paper with a patterned iron mask and a magnetic plate, followed by spraying the lacquer, and the hydrophobic barrier was created around the mask. Cardoso et al. positioned a magnetic mask on the filter paper and sprayed a scholar glue, which was subsequently subject to UV/Vis exposure. The hydrophobic barrier was formed after heating and cross-linking of the glue. Like stamping, a patterned mask is required. Cutting offers several merits such as high throughput, high resolution, simplicity, and no need of hydrophobic reagents; however, the devices suffer low mechanical strength and warpage [13]. In addition, the processing time depends on the complexity of the microfluidic design. Recently, the embossing technique was developed to construct paper-based microfluidics and its major characteristic is the use of mold inserts where either filter paper is deformed [31,32,33] or microstructures are created [34,35]. When two molds with complementary shapes were used, the filter paper was deformed such that an open channel was obtained and silanization was applied to the open channel, followed by sealing with a tape to form the closed channel [31]. Shin et al. compressed the nonwoven polypropylene sheet at high pressure such that the fibers at the embossed area were physically bonded together. This resulted in closure of the gaps between fibers, and the embossed area acted as the barrier to prevent the flow of fluids from leakage [32]. The channel can be engraved on the filter paper by using a ball-point pen filled with customized ink, which is left along the channel to hydrophobize the channel [33]. Juang et al. created protruded microstructures on the filter paper through embossing where wax was applied on the backside of the filter paper. The embossed filter paper was then subjected to heating, allowing the melted wax to diffuse into the filter paper. The melted wax was then cooled to solidify and the hydrophobic barrier was formed beneath the protruded microstructures, which then served as the microchannel for the fluid flow [34]. The processing time was approximately 1 min, which was further decreased to 10 s if both embossing and heating were performed simultaneously [35]. The advantages of the embossing technique are that it is simple, fast, and robust. Moreover, the processing time is not influenced by the design of microfluidics and there is great potential for mass production.

From the literature, it can be seen that the filter paper is the sole substrate material for paper-based microfluidics in most of the studies and there is no interrogation into utilization of regular printing paper, which is much more accessible and approximately three orders of magnitude cheaper compared to the cost of filter paper. Besides its low cost, the design of microfluidics can be generated readily by a commercially available printer. Spraying technique was adopted in this study to produce hydrophobic barrier around the mask, i.e., the design of the microfluidics, such that an additional metallic or magnetic mask or complicated setup was not required. The processing conditions such as printing times, spraying agent, spraying amount, wicking time, heating time, etc., were examined.

## 2. Materials and Methods

### 2.1. Fabrication and Characterization of Paper-Based Microfluidic Chips

**Figure 1:**
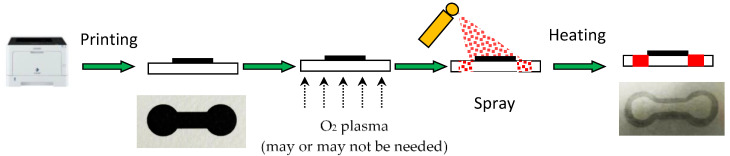
Schematics of spray on printed-paper process (not in scale).

Figure 1 shows the schematics of fabricating the paper-based microfluidic chips by spray on the printed paper. The dumbbell-shaped design was printed on the regular printing paper (Paperline gold 70p, Asia Pulp & Paper, Taipei, Taiwan) through a laser printer (AL-M220DN, Epson, Suwa-shi, Nagano, Japan). Different types of paper such as raw paper, tissue, paper towel, coated paper, simile paper, and watercolor paper were examined and they were either wrinkled after passing through the printer or the toner could not stick well to the paper. The diameter of the reservoirs is 8 mm and the width and length of the channel are 4 and 8 mm, respectively. After printing, the paper could be plasma treated for 60 s at 29.6 W (Plasma Cleaner, Harrick, Ithaca, NY, USA) if one would like to increase its hydrophilicity. Commercially available water repellent (Liquidoff, Dallas, TX, USA) or water wax (BlackPearl, Taoyuan, Taiwan) was used for spraying. In addition, a mixture of the polydimethylsiloxane (PDMS) base, curing agent, and ethyl acetate was prepared as an alternative spraying agent. The amount of spraying was characterized by first placing the paper on the balance, followed by spraying, and the weight was recorded. When using PDMS solution as the spraying agent, subsequent heating at 120 °C for 5 min was performed. To compare the flow behavior of solution wicking between the printing paper and the filter paper, strips with 5 mm in width and 35 mm in length were cut. One end of the strip was clamped and hung vertically upright and the other end was dipped into ink solution. The wicking distance from the bottom was recorded by digital camera. As for the flow in the microfluidic paper-based analytical devices (µPAD), it was placed under the microscope (SMZ-745T, Nikon, Tokyo, Japan) and the ink solution was dispensed in the reservoir at one end. The flow was recorded by computer software.

### 2.2. Glucose Detection

The fabricated paper-based microfluidic chips were used to perform glucose colorimetric detection. This is realized through enzymatic oxidation of glucose where glucose reacts with glucose oxidase (GOx) to produce hydrogen peroxide (H_2_O_2_). The H_2_O_2_ is then reduced to H_2_O by horseradish peroxidase (HRP) and potassium iodide (KI, colorless) is oxidized to iodine (yellow brown). Preparation of the glucose solution can be found elsewhere [34]. In brief, 1.5 µL of KI solution (0.03–0.6 M) was first dispensed at the detection zone. After drying under the ambient condition, 1.5 µL of HRP-GOx enzyme mixture at the ratio of 1:5 (or 3:5) was spotted at the detection zone. The concentrations of HRP and GOx were 0.2 and 0.4 mg/mL, respectively. A 15 µL measure of glucose solution with concentrations ranging from 1 to 50 mM (in pH 7.4 buffer) was then dispensed at the loading zone. The resultant color changes were observed under a microscope. The chip was then placed under ambient conditions for 15 min prior to taking a photo image and scanning. Quantification of the color response was carried out by using a commercially available scanner (Photosmart C4580, HP, Palo Alto, CA, USA) to capture the images of the detection zone, which were deconvoluted into red (R), green (G), and blue (B) components by computer software [34]. The intensity was taken as quantification of the color image.

## 3. Results and Discussion

### 3.1. Fabrication of the Paper-Based Microfluidic Chips by Spray on the Printed Paper

#### 3.1.1. Water Repellent and Water Wax as the Spraying Agent

**Figure 2:**
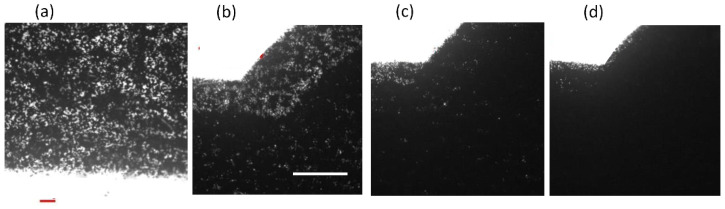
The toner coverage on the printed paper after printing (**a**) once, (**b**) twice, (**c**) three times, and (**d**) four times. Scale bar: 0.1 mm in (**a**) and 1 mm in (**b**). The scale bar in (**b**) also applies to (**c**,**d**).

**Figure 3:**
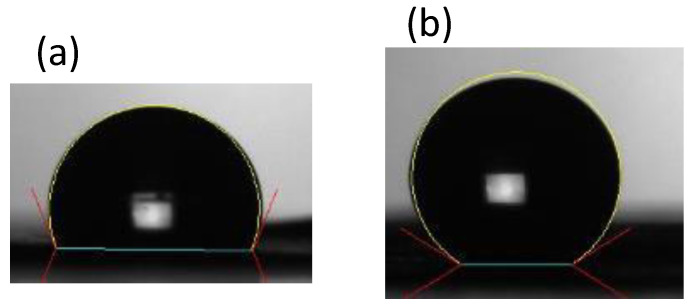
The sessile drop on the printing paper after spraying (**a**) water wax and (**b**) water repellent.

**Figure 4:**
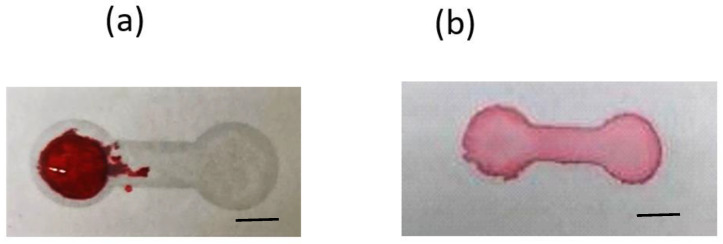
Dispensing the ink solution on the printed paper (**a**) without and (**b**) with O_2_ plasma treatment prior to spraying water wax. Scale bar: 5 mm.

For paper-based microfluidic chips, filter paper has been a nearly unanimous option to be used as the substrate material. There is no doubt that the filter paper possesses numerous attractive merits, however, porous material such as printing paper, which has much greater accessibility and is three orders of magnitude cheaper than filter paper, should be worthy of investigation. In addition, the microfluidic design can be printed out easily and rapidly. Our strategy is to utilize the printed pattern as the mask to block the hydrophobic spray to create the hydrophobic barrier around the pattern. Therefore, printing quality is the primary concern for successful development of our proposed technique. Figure 2 shows the printed pattern under the microscope. It can be seen that, with 600 dpi resolution, repetitive printing at least 3 to 4 times is required in order for the pattern to completely cover the printing paper. Note that overlap of the repetitively printed pattern was relatively good as the pattern was not enlarged and distorted. The commercially available water repellent and water wax were tested to spray on top of the printed paper. Although the blank paper became hydrophobic after spraying (Figure 3), it was found that the mask (i.e., the printed pattern) could not block the water repellent from penetrating through the paper. On the other hand, the water wax was blocked by the printed pattern. As the water wax was used, the processing parameters such as spraying distance, spraying amount, and time for the water wax to wick through the paper all played important roles in successfully fabricating the paper-based microfluidic chips. The optimized conditions were as follows: ~15 cm spraying distance, 1.5–2 g spraying amount, and 10–15 s for the wicking time. A clear boundary surrounding the channel was formed; however, there were some speckles inside the channel. When dispensing the ink solution in the loading zone, the ink solution did not wick through the channel as shown in Figure 4a.

It was hypothesized that the “speckles” were the wax agglomerate, which posed resistance to the flow even though the pores inside the filter paper were not completely filled by the wax. To resolve this issue, plasma treatment was applied prior to spraying and the ink solution wicked through the channel readily without leakage, as shown in Figure 4b.

#### 3.1.2. PDMS Mixture as the Spraying Agent

**Figure 5:**
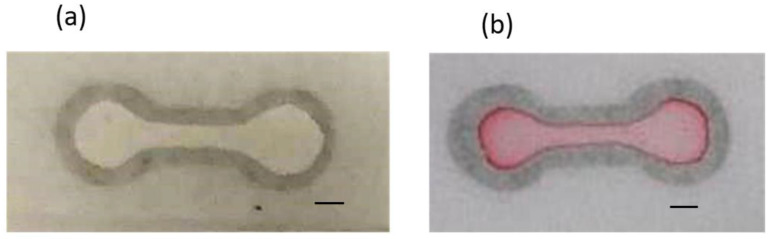
(**a**) The printed paper after spraying PDMS mixture. (**b**) The ink solution wicking through channel without leakage. Scale bar: 5 mm.

Although water wax as the spraying agent for this approach was successfully demonstrated, leakage was observed 1 h after the chip was fabricated. That is, the chips need to be used immediately after fabrication, rendering it not suitable for practical application. From the literature, the mixture of polydimethylsiloxane (PDMS) had been used to print and form the hydrophobic barrier in paper-based microfluidic chips [36]. In order to adapt to our technique, considerations need to be taken to ensure that the mixture not only is applicable to spraying but also contains enough content of PDMS for subsequent curing to form the barrier. Both hexane (used in the literature) and ethyl acetate were used to mix with PDMS. For hexane, it was found that the mixture dissolved the toner. On the other hand, the mixture of PDMS and ethyl acetate will not dissolve the toner and the ratio of PDMS to ethyl acetate for spraying was found to be 1:2.5. The optimized conditions for using the PDMS mixture to fabricate the paper-based microfluidic chips were as follows: repetitive printing four times, ~10 cm spraying distance, 5 s wicking time, 120 °C curing temperature, and 5 min curing time. Figure 5a shows the fabricated paper-based microfluidic chip. Note that the spraying amount plays little role when using the PDMS mixture provided repetitive printing for four times was applied. Furthermore, plasma treatment was not required if the printing paper was placed in the ambient for two weeks. Figure 5b shows that the ink solution wicked through the channel without leakage. The shelf life of the paper-based microfluidic chips as fabricated is at least 30 days.

### 3.2. Flow Characterization of Paper-Based Microfluidic Chips

**Figure 6:**
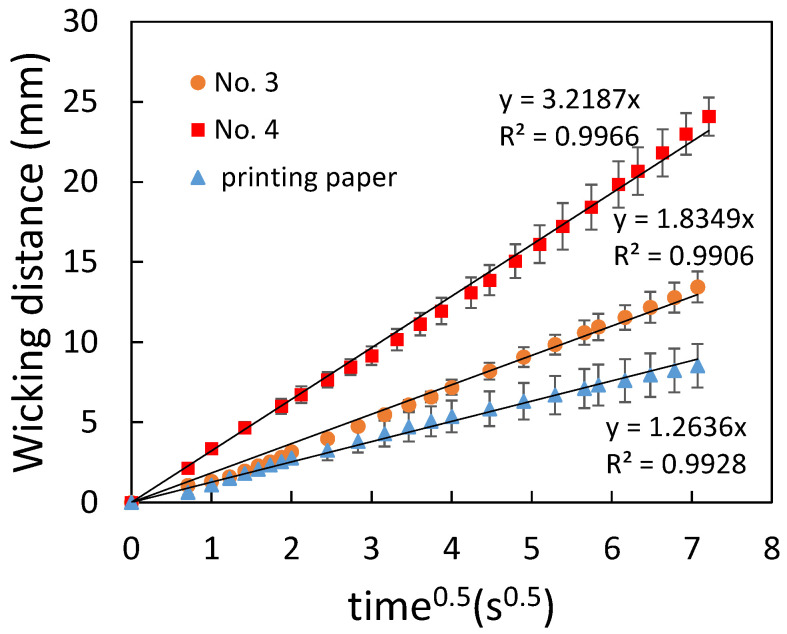
Comparison between the flow behavior of the filter papers and the printing paper.

**Figure 7:**
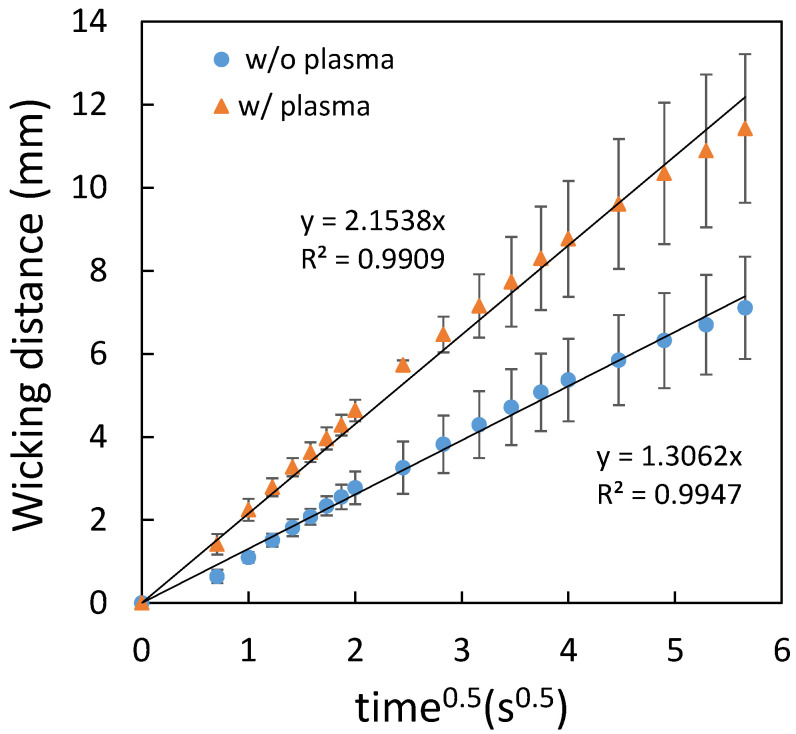
Comparison between the flow behavior of the printing paper with and without plasma treatment.

**Figure 8:**
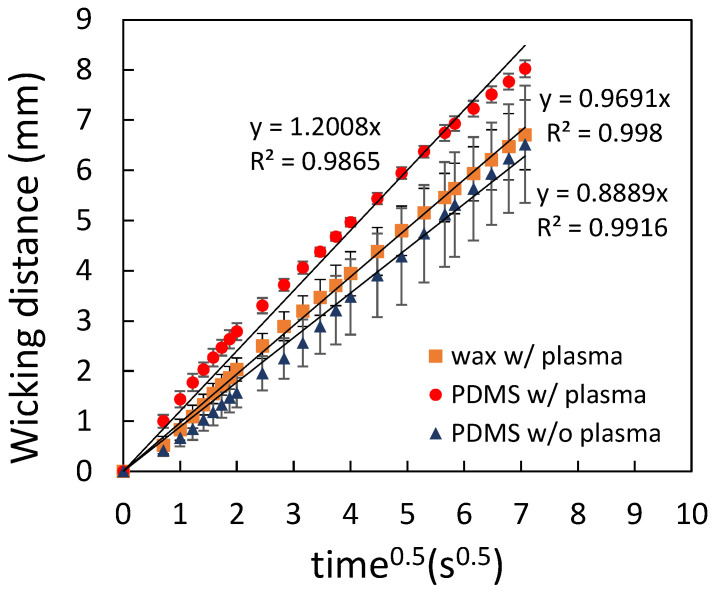
The flow behavior of the paper-based microfluidic chips fabricated by using different spraying agents, with and without plasma treatment.

To characterize the flow behavior, the wicking distance was measured and analyzed by Washburn’s equation as shown in the following:

(1) $$L=S\sqrt{t},\;S=\sqrt{\frac{\gamma Dcos\theta }{4\mu }}$$

where L is the wicking distance, *γ* is the liquid–vapor interfacial tension, *D* is the average pore diameter, θ is the contact angle for the three-phase system, *μ* is the liquid viscosity, and *t* is the time for liquid to wick through the distance L. Figure 6 shows the comparison of the flow behavior between the filter papers and the printing paper. It can be seen that a linear relationship was obtained for all cases. The error bars are the standard deviation of three independent measurements. Since the average pore diameter of Whatman No. 4 (20–25 μm) is larger than that of Whatman No. 3 (6 μm), the flow rate of the former was larger than the latter. As for the printing paper, the flow rate was smaller compared to that of the filter papers, indicating an even smaller average pore diameter of the printing paper. Note that the error bars in the measurement for the printing paper were slightly larger compared to those for the filter papers, implying a less-uniform porosity of the printing paper. When the printing paper was treated with plasma, the flow rate was increased as shown in Figure 7, which resulted from the increased hydrophilicity of the printed paper. Figure 8 shows the paper-based microfluidic chips fabricated by using different spraying agent with and without plasma treatment. As described previously, plasma treatment was required when using the water wax. It can be seen that the flow rate was similar when using either water wax (with plasma treatment) or the PDMS mixture (without plasma treatment). Although a larger flow rate could be obtained after plasma treatment, implementation of an additional procedure might not be beneficial in terms of cost and production rate.

### 3.3. Glucose Detection

**Figure 9:**
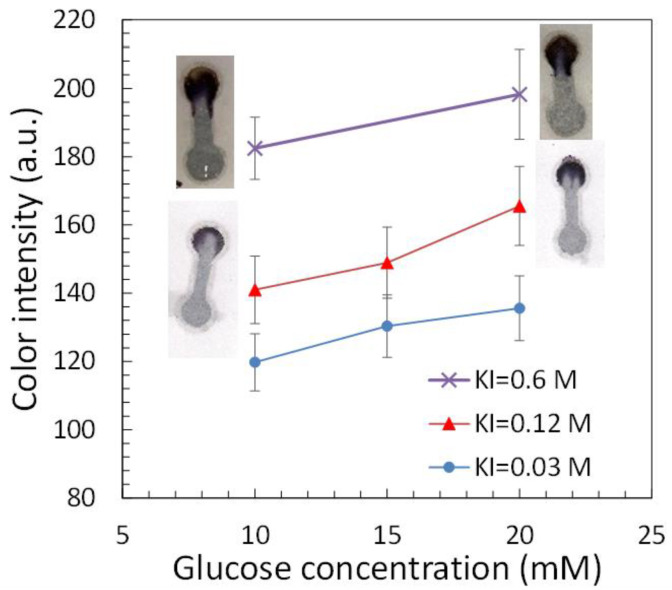
The color intensity for glucose detection at different KI concentrations. For microfluidics made of printing paper for diabetes monitoring, the KI concentration could be adjusted to 0.12 M such that the difference in glucose concentration in the blood samples from healthy people (≤10 mM) and diabetic patients (≥20 mM) can be distinguished.

Since glucose concentration in the blood for diabetic patients is larger than 10 mM [28], it would be desirable to be able to distinguish the difference of color intensity between 10 mM glucose concentration and higher. When using the 1:5 ratio of HRP to GOx and the 0.6 M concentration of potassium iodide (KI) solution as described in the literature, it was found that the color intensity was relatively strong for the glucose concentrations at 10 and 20 mM, as shown in Figure 9. The difference in the color intensity between these two concentrations was not conspicuous (about 15 a.u.). The glucose concentration larger than 20 mM also led to similar color intensity (data not shown). To resolve this issue, the KI concentration was decreased to 0.12 M and the color change was moderate, as shown in Figure 9. The difference in the color intensity between 10 and 20 mM was increased up to approximately 25 a.u. Further reducing the KI concentration to 0.03 M led to decrease in not only the overall color intensity but also the difference in the color intensity between 10 and 20 mM.

**Figure 10:**
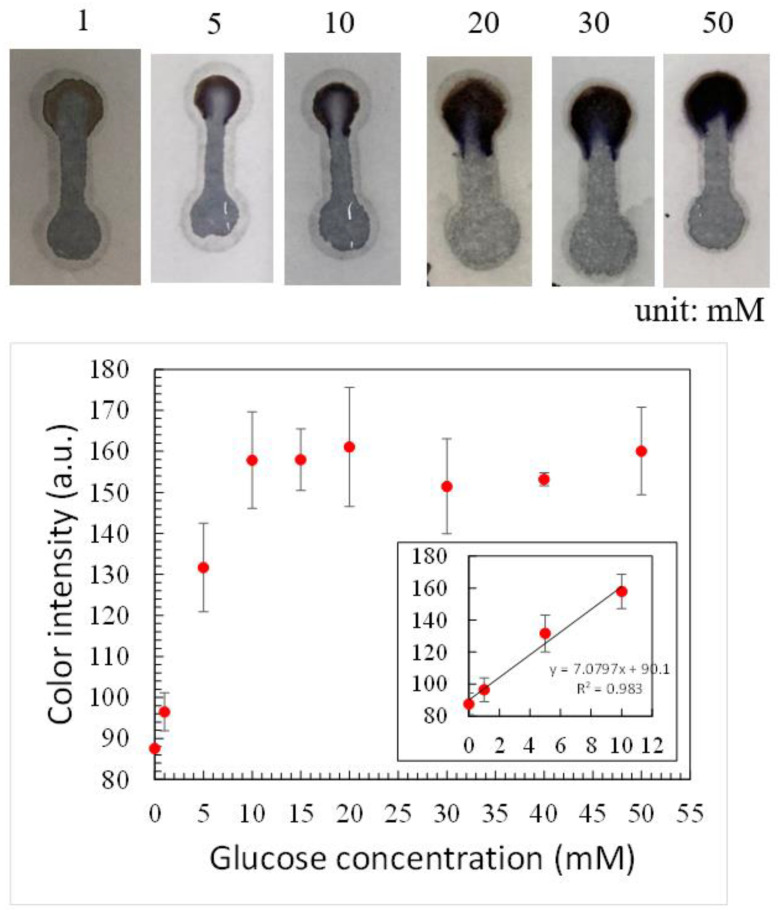
The color intensity for different glucose concentrations.

Non-invasive diabetes monitoring has been receiving great attention because it avoids the painful and invasive pricking process. Among various physiological fluids, urine is one of the most accessible body fluids and urine-based glucose detection has been extensively studied [37]. For healthy patients, the range of glucose concentration in the urine is 2.78–5.55 mM and it is greater than 5.55 mM for diabetic patients. To examine whether our device can be used to detect glucose concentration lower than 10 mM, the abovementioned operating conditions were applied and it was found that color intensity was very low when glucose concentration was around 5 mM. Therefore, KI concentration was increased back to 0.6 M while the ratio of HRP to GOx was changed to 3:5. Figure 10 shows the color intensity at different glucose concentrations. It can be seen that, at higher than 10 mM, the color intensity remained similar up to 50 mM. The minimum concentration detected was 1 mM and a linear relationship was obtained in the range between 1 and 10 mM, as shown in the inset. Using KI as the color indicator, a similar linear relationship between color intensity and glucose concentration was found in the literature [26,38,39,40,41] and the lowest detection concentration was around 5 mM. Note that slightly larger error bars were observed in the measurement of color intensity, which might result from various degrees of sample loss due to less uniform porosity in the printing paper as described previously, as sample loss could occur during fluid transport [42]. Nevertheless, the difference in color intensity between 1, 5, and 10 mM was distinguishable. That is, the glucose concentration either higher or lower than 5 mM can be measured. This indicates that our device has the potential to be used in urine-based diabetes monitoring.

Table 1 shows the comparison between commonly used techniques and our proposed method. It can be seen that substantial cost reduction with the paper substrate and its accessibility are the major advantages. Fabrication of microfluidic chips with complicated design can be achieved rapidly through printing.

## 4. Conclusions

In this paper, we proposed and demonstrated a novel approach by using printing paper in conjunction with spraying a mixture of PDMS and ethyl acetate to rapidly fabricate paper-based microfluidic chips. The greater accessibility and low cost of printing paper provide an attractive alternative for research and development in paper-based microfluidics. Glucose detection was carried out using paper-based microfluidic chips as fabricated and a linear relationship was obtained between 1 and 10 mM glucose concentration. The fabricated device is suitable for not only blood-based but also urine-based diagnostics for diabetes monitoring.

## Figures and Tables

**Table 1:** Comparison between commonly used techniques and proposed method.

	Processing Time (s)	Heating Time (s)	Heating Temp. (°C)	Equipment Cost (USD)	Substrate	Potential for Scalability
Solid wax printing	Depends on μPAD design	30–600	100–175	~500	Filter paper (~100 USD/m^2^)	Medium
Craft cutting	Depends on μPAD design	No	No	~250	Filter paper (100 USD/m^2^)	Medium
Spray on printed paper	~20 *	~300	120	~300	Printing paper (0.1 USD/m^2^)	High

* The processing time (i.e., the printing time) could be further decreased if a printer with higher dpi is used.

## Data Availability

Data available upon request.
